# Plant extract-mediated synthesis Cobalt doping in zinc oxide nanoparticles and their *in vitro* cytotoxicity and antibacterial performance

**DOI:** 10.1016/j.heliyon.2023.e19659

**Published:** 2023-08-30

**Authors:** Nouf M. Al-Enazi, Khawla Alsamhary, Fuad Ameen, Mansour Kha

**Affiliations:** a Department of Biology, College of Science and Humanities in Al-Kharj, Prince Sattam Bin Abdulaziz University, Al-Kharj, 11942, Saudi Arabia; b Department of Botany & Microbiology, College of Science, King Saud University, Riyadh, 11451, Saudi Arabia; c Antibacterial Materials R&D Centre, Huzhou Institute, Huzhou, Zhejiang, China

**Keywords:** Nanoparticles, Doped, Nanostructure, Cytotoxicity, Antimicrobial

## Abstract

In this research, zinc oxide (ZnO) nanoparticles doped with different percentages of produced cobalt using the green synthesis method. ZnO nanoparticles showed good cellular and microbial toxicity due to their high surface-to-volume ratio. Adding cobalt metal to the nanostructure can lead to the appearance of a new feature. To investigate the effect of adding cobalt metal, synthesized ZnO nanoparticles containing 3 and 6% cobalt were synthesized using plant extract. The resulting nanostructures were characterized by a Raman spectroscopy, UV–Visible spectrometer, X-ray diffraction, and Field emission scanning electron microscopy. Ultimately, the synthesized samples' cytotoxicity and antimicrobial tests were performed. XRD confirmed the formation of a hexagonal wurtzite ZnO structure. XRD and electron imaging showed that doping resulted in a decrease in average crystal size. The results showed that with cobalt doping, the particle size decreased slightly. The cytotoxicity and antimicrobial effects results showed that in all three studies, cobalt doping leads to an increase in the toxicity of this nanostructure compared to non-doped nanoparticles.

## 1 Introduction

Infectious diseases and cancer are among the most critical problems that limit living organisms' health worldwide [[1], [2], [3]]. Reports indicate that millions of deaths are caused by these diseases every year [4]. Infectious diseases and cancer seriously threaten human health [5]. Despite the tremendous advances in medicine and the provision of numerous treatment solutions, due to non-specificity, severe side effects, the emergence of resistance, or change like the disease agent, the need to develop and discover an effective and appropriate treatment for these diseases is felt [[6], [7], [8]]. Adequate and appropriate treatment is desired by creating a system for targeted drug delivery to the tissue without side effects or a specific effect on the disease agent [9,10].

Nanotechnology is a new global science, rapidly growing and widely considered in various fields, including engineering and medicine [[11], [12], [13]]. It has been shown that nanoparticles, due to their extraordinary properties such as small size [14], optical properties [15], conductivity, porosity, and high surface-to-volume ratio [16], can be used to design new and innovative treatment methods for all kinds of diseases [17,18].

Cobalt is a ferromagnetic metal with an atomic number 27 and specific gravity of 8.9. Cobalt metal has two crystallographic structures: fcc and hcp. Cobalt is an essential element with many oxidation states. Halogens and sulfur attack this element. Heating it in oxygen produces Co_3_O_4_, which loses oxygen at 900 °C to form cobalt (II) oxide [19]. Nanoparticles containing cobalt magnetic parts have been considered due to their magnetism [20]. The property of magnetism has caused the application of these structures in medicine, industry, sensors, and magnetic memories [21]. With the development and production of new magnetic structures, the medical applications of these structures have been significantly affected and are being developed. Diagnosis and treatment of disease agents and intelligent drug delivery are among these magnetic structures' most widely used areas [22,23]. One of the most important types of these structures is cobalt-based nanostructures. Magnetic nanostructures that can be intelligently guided to the desired location by magnetic fields are used as carriers of drugs or genetic materials [24]. Therefore, they can be used in the treatment of infectious diseases and especially cancer-related diseases [25]. Magnetic nanoparticles are used as contrast enhancement agents in magnetic resonance imaging [26]. Considering the new applications of nanostructures, researchers are looking for ways to reduce the economic and environmental challenges in producing these nanostructures and make the process of making these nanostructures simpler and more efficient [27,28]. The electrochemical and sol-gel [29] methods were reported as influential among the various methods presented for making nanoparticles. Although these methods are efficient, they depend on expensive equipment and expensive chemicals, which are not only economically unattractive but also limit the health of the environment due to the high energy consumption and harmful chemicals [[30], [31], [32]]. The particle stability, and industrial scale production are difficult in synthesis methods of the microwave [33], electrochemical [34] and sol-gel methods. The use of plants to synthesize nanostructures is essential due to their abundance and compatibility with the environment [[35], [36], [37]]. Plant extracts or biomass is a suitable alternative for physical and chemical nanoparticle synthesis methods [[38], [39], [40]].

Therefore, in this study, to provide an efficient, economical, new, and environmentally friendly plant synthesis method, cobalt doped zinc oxide nanostructures for use in research projects in medicine.

## 2 Materials and methods

### 2.1 Reagents

HCl (Hydrochloric acid, Merck), DMSO (Dimethyl sulfoxide, Sigma), DMEM culture medium (Sigma), fetal bovine serum (FBS) and l-glutamine (Merck) are used.

### 2.2 Prepare of Co-doped ZnO-NPs

The Co-doped ZnO-NPs were synthesized using plant (*Prosopis fractal*) extract. A soaking procedure did the extraction. To prepare for the extraction, the dried leaves of these plants are powdered. The obtained powder (10 gr) was added to 30 mL of distilled water. For the synthesis, first, the prepared extract was diluted; for this purpose, 10 mL of the extract was added to 100 mL of double distilled water, and then zinc nitrate was added to it and thoroughly stirred for half an hour using a heater stirrer to be resolved entirely. Then, the pH of the sample was adjusted to 12 by adding NaOH. The resulting reaction mixture was dried and calcined at 500° (Celsius). Then, the obtained samples were zinc oxide nanoparticles. The Co-doped ZnO-NPs with Zn_1-x_Co_x_O (X = 0, 0.3 and 0.4 mol) formula were prepared using a similar procedure in which Co(NO_3_)_2_.6H_2_O was added along with zinc nitrate at mentioned stoichiometric ratios.

### 2.3 Co-doped ZnO-NPs characterization

A Philips X-ray Diffractometer model −1800PW with CuKα monochromatic radiation source and with a wavelength of λ = 1.54 Å was used to prepare X-ray diffraction (XRD) patterns. Diffraction angle values were recorded at 2θ in the range of 10 to 80°. Raman spectroscopy was performed to study and detect the integration of impurity and lattice structure defects. Field emission scanning electron microscopy (FESEM) was used to prepare images of nanoparticles and study the size and surface morphology [41].

### 2.4 Cell toxicity assay

Cancer cells (MCF7) prepared and cultured in appropriate culture medium (RPMI) with 10% fetal bovine serum (FBS) and 1% streptomycin antibiotic at 37 °C and 5% CO_2_. Cancer cells with 5 × 10^4^ concentration were prepared and added to each well of the 96-well plate. Then, different concentrations of Co-doped ZnO-NPs (1–100 μg/mL) were treated for 72 h at a temperature of 37 °C. Then, an MTT reagent with a concentration of 5 mg/mL was used to evaluate the toxicity effect of Co-doped ZnO-NPs on cancer cells.

The MTT assay is one of the most common methods of investigating cell toxicity, which is used with the colorimetric method to evaluate the degree of toxicity of substances on cell viability. This issue is investigated by measuring the power of reducing yellow tetrazolium dye (MTT) after penetration into living cells by mitochondrial succinate dehydrogenase enzyme and converting it into purple formazan crystals. After dissolving these crystals with the help of DMSO, the amount of absorption of this solution indicates the viability of the cells. The cytotoxicity of Co-doped ZnO-NPs was examined using the MTT test to find the effect of toxicity in concentrations from 0 to 100 μg/mL) within 24 h. After 24 h of the proximity of Co-doped ZnO-NPs with cells, 5 mg/mL of MTT was added to each well separately, then the cells were transferred to 37 °C incubator for 4 h. In the next step, the culture medium on the cells was slowly removed, and 100 mL of DMSO were added to each well. After the complete dissolution of the formazan crystals, the absorbance of the solutions of each well was recorded using an ELISA device. A concentration of Co-doped ZnO-NPs that limits the growth of 50% of cells was determined as IC_50_ [42,43]^.^

### 2.5 Antibacterial properties test

The Müller–Hinton agar (38 gr) and Nutrient agar (28 gr) were dissolved in 1000 mL of deionized distilled water to prepare bacteria culture media. Then it was sterilized in an autoclave for 15 min at a temperature of 121° Celsius. At last, it was poured into sterile plates. To ensure the absence of any contamination, they were placed in an incubator at 37 °C for 24 h. In order to prevent the entry of any microbial contamination into the culture medium, all these steps were performed next to the flame and under sterile conditions. The diameter of the halo of non-growth of bacteria was investigated by different of Co-doped ZnO-NPs on *Staphylococcus aureus* and *Escherichia coli* cultured for 24 h at 37 °C by welling in agar or spreading wells in agar. For this purpose, 24 h after making a well on the plate and transferring 25 mL of Co-doped ZnO-NPs in different concentrations into each well, the diameter of the halo of non-growth of bacteria by Co-doped ZnO-NPs on the plates was measured and reported in centimeters with a precise ruler. In the MIC evaluation, first, we dissolved 0.2 g of Co-doped ZnO-NPs in 1 mL of sterile deionized water and prepared the original stock. In the next step, after preparing the concentrations of 0–100 μg/mL, we took 50 mL from the leading stocks of the desired substance using a sterile sampler. We took 50 mL from the leading stocks of the desired substance. We added it to 1000 mL of Mueller Hinton Broth culture medium. To perform the test, the serial dilution method was used. After completing this step, 100 mL of the prepared bacteria were added to all the tubes except the control tube, which contained the liquid culture medium and the primary test substance. An additional tube containing only the liquid culture medium and the desired bacteria was considered a control. Then, all the tubes were transferred to a shaker incubator at 37 °C and 150 rpm and heated for 24 h. After this period, the tubes were removed from the incubator, and their MIC was measured by direct eye observation and concentration measurement with a nanodrop device. All the above steps were performed under sterile conditions, under a laminar hood, and next to the flame [[44], [45], [46]].

## 3 Results and discussion

**Fig. 1:**
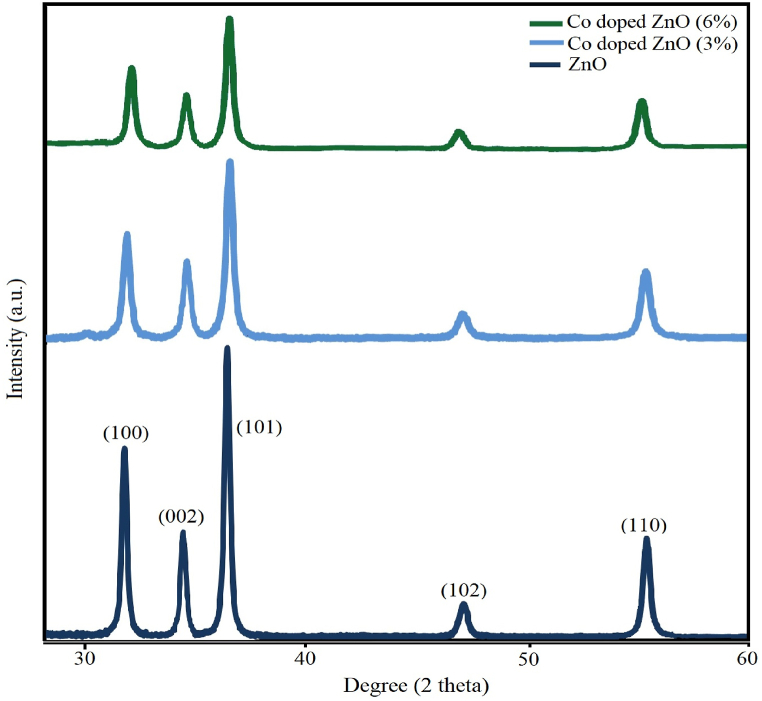
The XRD patterns of ZnO and Co-doped ZnO NPs with concentrations of 3 and 6% of cobalt.

The XRD patterns of ZnO and co-doped ZnO samples with different concentrations of cobalt (3 and 6%) are shown in Fig. 1. The peaks observed in the XRD patterns are related to the (100), (002), (101), (102), (110), (103), (200), (112), (202) planes, confirmed forming a hexagonal Wurtzite structure of ZnO that was similar with the previous reported results [[47], [48], [49], [50]]. The absence of diffraction peaks from other Co phases confirms that Co ions were placed in the ZnO lattice instead of Zn ions without changing the wurtzite structure. Scherrer's equation was used to determining crystallite sizes of resulting nanoparticles. Obtained. The crystallite sizes of pure and Co-doped-NPs were 28.7 and 25.17 nm, respectively. As the percentage of Co in the structure increased, the intensity of the peaks decreased, indicating a decrease in crystallinity. Doping led to the formation of defects and stress in the ZnO crystal structure. The results of crystallite size analysis using Scherer's formula showed that cobalt doping leads to a decrease in crystallite size, and the increase in doping percentage and decrease in size has a direct relationship. An increase in the doping percentage of cobalt ions leads to a further decrease in the peristaltic size. These results indicate that cobalt ion interferes with the growth of ZnO nanocrystals.

**Fig. 2:**
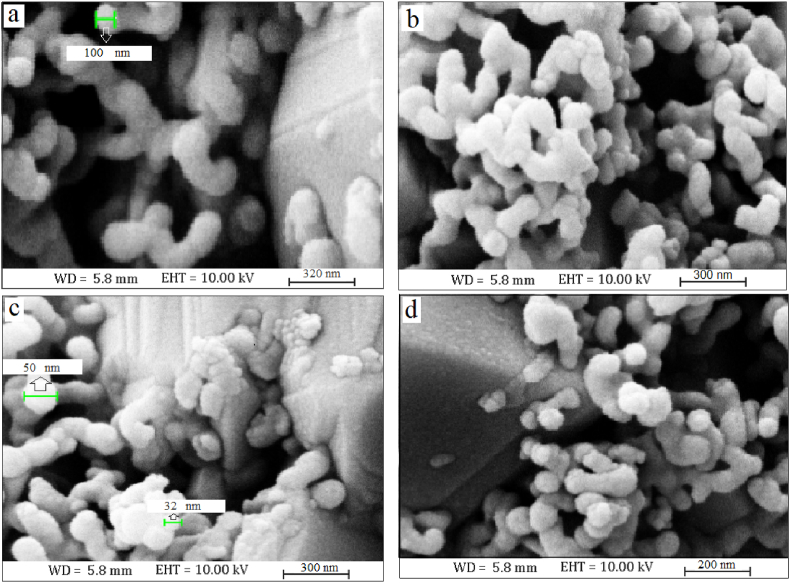
FESEM of ZnO (a) and Co-doped ZnO NPs with concentrations of 3 (b and c) and 6% (d) of cobalt.

FESEM revealed the morphology of irregular spherical Co-doped ZnO NPs (Fig. 2). According to the FESEM results, it is clear that cobalt doping has reduced the nanostructures' size.

**Fig. 3:**
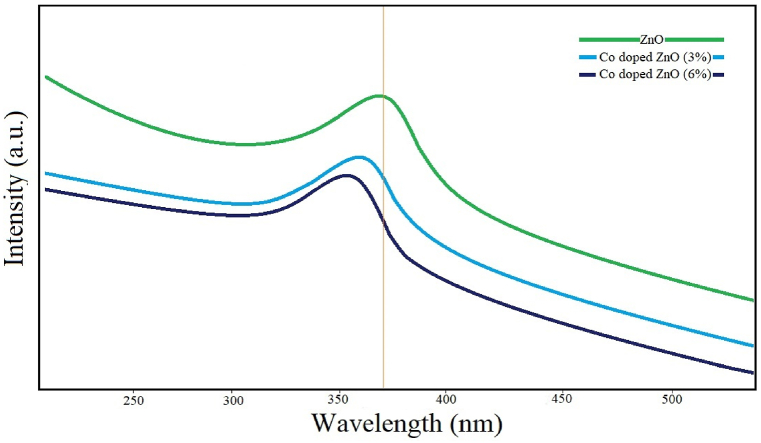
The UV–Vis absorption of ZnO (a) and Co-doped ZnO NPs with concentrations of 3 and 6% of cobalt.

The UV–Vis absorption spectrum of Co-doped ZnO NPs is in the range of 200–550 nm. The spectrum of undoped ZnO showed an absorption band of about 381 nm, which is characteristic of the absorption of ZnO (Fig. 3). Co-doping led to a shift towards lower wavelengths, possibly due to the presence of defects due to cobalt that was similar with the previously reported results [51].

**Fig. 4:**
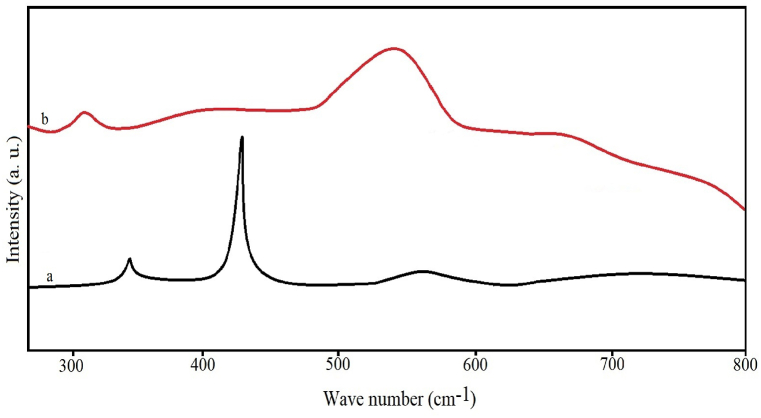
Raman spectra of ZnO (a) and Co-doped ZnO NPs (b).

Raman spectrum of pure and Co-doped are shown in Fig. 4. In the Raman spectrum analysis, the change of charge transfer due to impurity ions in the doped NPs leads to a change in the optical Raman spectrum. The peak at 438 cm^−1^ can be assigned as E 2 of ZnO, related to the wurtzite crystal structure. Raman spectrum of ZnO and Co-doped ZnO NPs shows the creation of intrinsic defects and the formation of different structural defects. The change in peak intensity occurred due to Co doping into the ZnO lattice. Cobalt doping in the structure caused a defect, leading to the peak splitting. Results showed the peak shift towards the low wave number after doping as the Co ions increased, similar to the previously reported results [52].

**Fig. 5:**
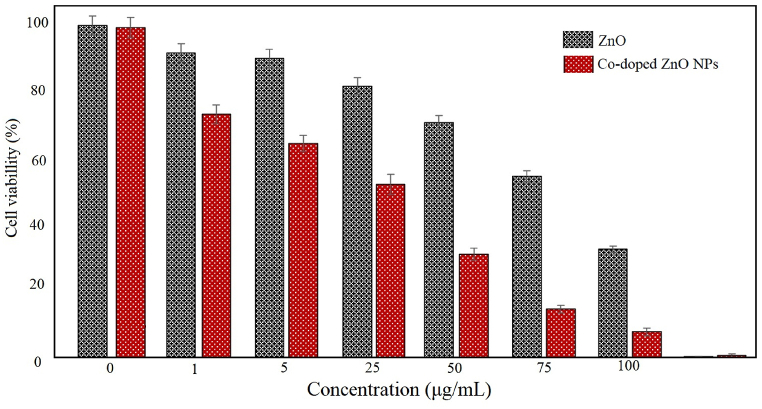
Cytotoxic activity of ZnO and Co-doped ZnO NPs.

The present study tested the cytotoxicity effect of ZnO and Co-doped ZnO NPs against MCF7 cancer cells. The cytotoxicity activity for each concentration was repeated three times (Fig. 5). The IC_50_ of ZnO was at 100 μg/mL. The IC50 of ZnO NPs doped with Cobalt increased significantly (50 μg/mL).

The antibacterial effect of ZnO nanoparticles doped with Cobalt increased significantly. The IC_50_ of ZnO for both bacteria was 5 μg/mL. The IC_50_ of ZnO for both bacteria were 1 μg/mL. Co-doping has led to a decrease in the size of ZnO nanoparticles, which can cause better penetration and solubility of this nanostructure. It hence may have increased the toxicity of the Co-doped ZnO NPs.

The present study tested the antibacterial effect of ZnO and Co-doped ZnO NPs against *Staphylococcus aureus* and *Escherichia coli*. The antibacterial activity for each concentration was repeated three times. The antimicrobial activity of the Co-doped ZnO NPs was also proven by observing and measuring the zone of inhibition of the bacteria growth. Antibacterial activity of ZnO and Co-doped ZnO NPs has been shown in Table 1. The highest antimicrobial effect of ZnO NPs was against *S. aureus* (12 ± 0.5), and the lowest effect was against *E. coli* (10 ± 0.5). The highest antimicrobial effect of Co-doped ZnO was against *S. aureus* (16 ± 0.5), and the lowest effect was against *E. coli* (13 ± 0.5).

**Table 1:** Antibacterial activity of ZnO and Co-doped ZnO NPs Zone of Inhibition.

Nanoparticles	*Staphylococcus aureus s*	*Escherichia coli*
	Zone of Inhibition (mm)	
Pure ZnO	12 ± 0.5	10 ± 0.5
Co-doped ZnO	16 ± 0.5	13 ± 0.5

## 4 Conclusion

ZnO nanoparticles doped with cobalt in different percentages (3 and 6%) were synthesized using plant extract. The effect of doping on morphology, cytotoxicity, and antibacterial activities has been investigated. XRD confirmed the formation of a hexagonal wurtzite ZnO structure. XRD and electron imaging showed that doping resulted in a decrease in average crystal size. Cytotoxicity and antibacterial studies against *S. aureus* and *E. coli* showed that in both cellular and bacterial toxicity studies, nanoparticles doped with cobalt had more toxic effects than non-doped ZnO nanoparticles. This indicated the increase in toxicity of nanoparticles due to the addition of cobalt in the structure of ZnO nanoparticles.

## Author contribution statement

Nouf M Al-Enazi: Wrote the paper; Conceived and designed the experiments; Performed the experiments; Analyzed and interpreted the data.

Khawla Alsamhary: Wrote the paper; Analyzed and interpreted the data.

Fuad Ameen: Wrote the paper; Conceived and designed the experiments; Contributed reagents, materials, analysis tools or data.

Mansour Kha: Wrote the paper; Performed the experiments.

## Data availability statement

No data was used for the research described in the article.

## Declaration of competing interest

The authors declare that they have no known competing financial interests or personal relationships that could have appeared to influence the work reported in this paper.
